# The effect of epigallocatechin gallate on laying performance, egg quality, immune status, antioxidant capacity, and hepatic metabolome of laying ducks reared in high temperature condition

**DOI:** 10.1080/01652176.2023.2280041

**Published:** 2023-11-15

**Authors:** Yang Liu, Xu Zhang, Yaling Yao, Xuan Huang, Chuang Li, Ping Deng, Guitao Jiang, Qiuzhong Dai

**Affiliations:** a Hunan Institute of Animal Husbandry and Veterinary Medicine, Changsha, China; b Huaihua Animal Husbandry and Aquatic Transaction Center, Huaihua, China

**Keywords:** Epigallocatechin gallate, laying ducks, production performance, antioxidant, hepatic metabolomics, high temperature

## Abstract

Epigallocatechin gallate (EGCG) is a main component in green tea extract, which possesses multiple bioactivities. The present research studied the effects of EGCG on the laying performance, egg quality, immune status, antioxidant capacity, and hepatic metabolome of *Linwu* laying ducks reared under high temperature. A total of 180 42-w-old healthy *Linwu* laying ducks were allocated into control or EGCG-treated groups. Each treatment had 6 replicates with 15 ducks in each replicate. Diets for the two groups were basal diets supplemented with 0 or 300 mg/kg EGCG, respectively. All ducks were raised in the high temperature condition (35 ± 2 °C for 6 h from 10:00 to 16:00, and 28 ± 2 °C for the other 18 h from 16:00 to 10:00 the next day) for 21 days. Results showed that EGCG increased the egg production rate (*p* = 0.014) and enhanced the immunocompetence by improving serum levels of immunoglobulin A (*p* = 0.008) and immunoglobulin G (*p* = 0.006). EGCG also fortified the antioxidant capacity by activating superoxide dismutase (*p* = 0.012), catalase (*p* = 0.009), and glutathione peroxidase (*p* = 0.021), and increasing the level of heat-shock protein 70 (*p* = 0.003) in laying ducks’ liver. At the same time, hepatic metabolomics result suggested that EGCG increased the concentration of several key metabolites, such as spermidine (*p* = 0.031), tetramethylenediamine (*p* = 0.009), hyoscyamine (*p* = 0.026), β-nicotinamide adenine dinucleotide phosphate (*p* = 0.038), and pantothenic acid (*p* = 0.010), which were involved in the metabolic pathways of glutathione metabolism, arginine and proline metabolism, β-alanine metabolism, and tropane, piperidine, and pyridine alkaloid biosynthesis. In conclusion, 300 mg/kg dietary EGCG showed protection effects on the laying ducks reared in high temperature by improving the immune and antioxidant capacities, which contributed to the increase of laying performance of ducks. The potential mechanism could be that EGCG modulate the synthesis of key metabolites and associated metabolic pathways.

## Introduction

Due to the popularity of intensive rearing systems and climatic issue of global warming, heat stress is considered as a big challenge to poultry industry due to its detrimental effects on health, performance, and welfare (Lara and Rostagno 2013). The economic loss in poultry industry caused by heat stress reaches over $165 million annually (Wasti et al. 2020). The optimal growing temperature range for birds older than 28 d is between 18 and 23 °C, and exposure to environment temperature higher than 25 °C can induce acute or chronic heat stress to the birds, depending on the exposed time period (Vandana et al. 2021). Laying birds in high temperature condition experienced physiological changes such as increased respiration rate, increased blood pH, and reduced feed intake, which ultimately lead to reduction in egg production performance and egg quality (Barrett et al. 2019).

Apart from the common physical strategies combating high temperature, such as exhaust fans, cooling pads, and sprinkling cool water, phytochemicals with the characteristics of safety and multiple bioactivities became another solution for poultry as new feed additives (Akbarian et al. 2016). Epigallocatechin gallate (EGCG) is the primary component of green tea extract. It belongs to polyphenols, and possesses strong antioxidant and anti-inflammatory capacities (Kim et al. 2018; Hu et al. 2019; Ma et al. 2021; Wang et al. 2021). Previous studies stated that EGCG improved the growth performance of heat-stressed broilers by alleviating oxidative damage (Luo et al. 2018), and relieved oxidative stress in heat-stressed quails by modulating the hepatic nuclear transcription factors (Sahin et al. 2010). Additionally, supplementing laying hens’ diets with green tea extract diet was reported improve egg production, feed efficiency, egg white quality and the antioxidant capacity of eggs (Wang et al. 2017, 2018; Zhu et al. 2020). However, few research is available regarding to the effect of EGCG on laying ducks, and the mechanism of the effects of EGCG was vague. Therefore, this study was designed to investigate the effects of dietary EGCG on production performance, antioxidant capacity, and immune status of *Linwu* laying ducks reared in high temperature, and attempted to interpretate the potential mechanism by using metabolomics.

## Material and methods

The animal experiment of this study was approved by Hunan Institute of Animal Husbandry and Veterinary Medicine (Changsha, Hunan, China) under protocol code AHVM20220801.

### Experiment design and diets

A total of 180 43-w-old healthy *Linwu* laying ducks were selected for this study, which were transferred from commercial farm to two-layer plastic cages (35 cm × 40 cm × 35 cm) with rollaway to egg-collecting cradles. The ducks were housed individually in the cages and were assigned to 2 groups to make sure that the egg production rate was similar between the two groups. The facility was maintained at 35 ± 2 °C for 6 h (10:00 till 16:00) using heaters, and 28 ± 2 °C (16:00 till 10:00 the next day) in the 21-d-trial. The wet pad was operated to cool down the temperature when necessary. The relative humidity of the facility was kept between 60 and 65%. Each group had 6 replicates with 15 ducks per replicate. Ducks in the two groups were provided with basal diet supplemented with 0 (CON group) or 300 mg/kg EGCG (EGCG group), and free access to water and diet was provided throughout the trial. EGCG used in the study was in white powder with purity of 98%, which was purchased from Huilin Bio-Tech Co., Ltd (Xi’an, China). The trail last 21 days, and the composition and nutrient levels of the basal diet are shown in Table 1.

**Table 1.** Composition and nutrient levels of basal diet (air-dry basis, %).

Ingredients	Content	Nutrient levels^c^	Content
Corn	31.94	Metabolic energy (MJ/kg)	10.24
Soybean meal	26.88	Crude protein	18.00
Wheat	16.00	Linoleic acid	1.57
Rice bran meal	10.00	Crude fiber	6.72
Wheat middling	2.00	Ether extract	2.95
Soybean oil	1.00	Lysine	0.92
CaHPO_4_	1.00	Methionine	0.48
Limestone	9.00	Threonine	0.64
NaCl	0.30	Tryptophan	0.20
Mildewproof agent	0.15	Calcium	3.54
l-Lysin (78%)	0.13	Total phosphate	0.67
dl*-*Methionine (98%)	0.13		
Threonine (98%)	0.13		
Zeolite powder	1.06		
Vitamin complex for poultry^a^	0.03		
Choline chloride (50%)	0.10		
Trace elements^b^	0.10		
Phytase (10,000 U/g)	0.02		
Probiotics (10^8^/g)	0.01		
Complex enzyme	0.02		
Total	100.00		

^a^ Vitamin complex for poultry provides the following per kg of diets: VA 12000 IU, VB_1_ 4 mg, VB_2_ 10 m, VB_5_ 40 mg, VB_6_ 6 mg, VB_12_ 0.03 mg, VD_3_ 2000 IU, V_E_ 20 IU, VK_3_ 1 mg, biotin 0.15 mg, folic acid 0.6 mg, *D*-pantothenic acid 60 mg, and nicotinic acid 50 mg.

^b^ Trace element provides the following per kg of diets: Cu (as copper sulfate) 10 mg, Fe (as ferrous sulfate) 60 mg, Mn (as manganese sulfate) 90 mg, Zn (as zinc sulfate) 90 mg, I (as potassium iodide) 0.50 mg, and Se 0.4 mg.

^c^ Nutrient levels and ME were calculated values.

### Production performance

During the trial, number and weight of produced eggs, amount of feed intake, and mortality of ducks were recorded daily on replicate basis. And the number of unqualified eggs, which had soft shell, broken shell, and irregulated shape, was recorded. Egg production rate, qualified egg rate, average egg weight, average daily egg yield, feed to egg ratio (F/E), and mortality for each replicate were calculated to represent the production performance of laying ducks at the end of trial based on the following formulas:

$$\begin{array}{l} \text{Egg}\;\;\text{production}\;\;\text{rate}\;\;\text{=}\;\;\text{number}\;\;\text{of}\;\;\text{total}\;\;\text{produced}\;\;\text{eggs/}\\ \text{number}\;\;\text{of }\;\text{days}\;\times \;\text{100\%} \end{array}$$

$$\begin{array}{l} \text{Qualified}\;\;\text{egg}\;\text{rate}\;\;\text{=}\;\;\text{number}\;\;\text{of}\;\;\text{qualified}\;\;\text{eggs/}\\ \text{number}\;\;\text{of}\;\;\text{total}\;\\ \;\text{produced}\;\;\text{eggs}\;\times \;\text{100\%} \end{array}$$

$$\begin{array}{c} \text{Average}\;\;\text{egg}\;\;\text{weight}\;\;\text{=}\;\;\text{weight}\;\;\text{of}\;\;\text{total}\;\;\text{produced}\;\;\text{eggs/}\\ \text{ number}\;\;\text{of}\;\;\text{total}\;\;\text{produced}\;\;\text{eggs} \end{array}$$

$$\begin{array}{c} \text{Average}\;\;\text{daily}\;\;\text{egg}\;\;\text{weight}\;\text{=}\;\text{average}\;\;\text{egg}\;\;\text{weight/}\\ \text{ number}\;\;\text{of}\;\;\text{days} \end{array}$$

F/E = total  feed  intake/number  of  total  produced  eggs

$$\begin{array}{c} \text{Mortality}\;\text{=}\;\text{number}\;\;\text{of}\;\;\text{died}\;\;\text{ducks/}\\ \text{ number}\;\;\text{of}\;\;\text{ducks}\;\;\text{at}\;\;\text{the}\;\;\text{beginning}\;\times \;\text{100\%} \end{array}$$

### Egg quality

At the day 21, 4 eggs were randomly selected from each replicate (24 eggs for each group) for quality evaluation, represented by parameters of eggshell strength, egg shell thickness, yolk color, and Haugh unit (HU). Eggshell thickness was measured by an eggshell thickness gauge (ESTG-1, Herzliya, Israel). Eggshell strength was measured by an egg force reader (EFR-01, Herzliya, Israel). Eggshell thickness was the average thickness of the sharp end, blunt end, and eqator end of eggshell after the interrior membrance being removed. Yolk color and HU were determined by an egg multitester (EMT-7300, Loimaa, Finland). The associated methods were referred to an earlier paper (Barrett et al. 2019).

### Serum immune status

Two ducks were randomly chosen from each replicate at the end of trial for sample collection (12 ducks for each group). Blood was collected from wing vein with vaccum tubs, and centrifuged at 3000×*g* for 10 min to obtain serum. Immune status related parameters including immunoglobulin A (IgA), immunoglobulin G (IgG), and immunoglobulin M (IgM) were measured with commercial assay kits (Jianglai Biotechnology Co., Ltd., Shanghai, China) and an automated fluorescence instrument (Multiskan^TM^ Skyhigh, Thermo Fisher Scientific, Waltham, USA).

### Hepatic antioxidant status

After blood collection, the sample ducks were slaughtered by cervical dislocation. A piece of 0.3 g liver tissue was taken from each sample duck (12 samples for each group) and homogenized with cold saline (tissue weight: saline volume = 1: 9) using a homogenizer (Tekmar, Cincinnati, USA), following by centrifugation at 4000×*g* for 15 min in 4 °C condition. The supernatants were carefully collected for antioxidant capacity test. The hepatic antioxidant capacity related parameters included level of heat shock protein 70 (Hsp-70) and activities of superoxide dismutase (SOD), malonaldehyde (MDA), catalase (CAT), total antioxidant capacity (T-AOC), and glutathione peroxidase (GSH-Px). These parameters were measured with commercial assay kits (Jianglai Biotechnology Co., LTD, Shanghai, China, item code: H264-2 for Hsp-70 kit, A001-1 for SOD kit, A003-1 for MDA kit, A007-2 for CAT kit, A015-2 for T-AOC kit, A005-1 for GSH-Px kit) and an automated fluorescence instrument (Multiskan^TM^ Skyhigh, Thermo Fisher Scientific, Waltham, USA).

### Hepatic metabolomic analysis

A portion of liver tissue from each sample duck (12 samples for each group) were collected for metabolomic analysis test, following the method described previously (Cai et al. 2015). Briefly, 25 mg sample was removed to a 1.5 mL EP tube, and 500 μL extract solution (methanol: acetonitrile: water = 2: 2: 1) was added into the tube. Then the mixture was ultrasonic shaken in ice baths for 1 h and placed at −20 °C for 1 h, following by homogenization at 14,000 g for 20 min at 4 °C condition. Then the supernatant was retrieved and concentrated to dryness in vacuum for further LC-MS/MS analysis.

As a part of the system conditioning and quality control process, a pooled quality control (QC) sample was prepared by mixing equal volumes of all samples. An ultra-performance liquid chromatography (UPLC) system (1290 Infinity LC, Agilent Technologies, Santa Clara, USA) and ACQUITY UPLC BEH Amide 1.7 μm column (Waters, Drinagh, Ireland) were used to identify the chromatographic separations. The sample injection volume was set at 2 μL and the flow rate was 0.5 mL/min. The column temperature was maintained at 40 °C and all samples were store at 4 °C during the period of analysis. The mass spectrometric data was collected using a ProteoWizard MSConvert and processed using XCMS for feature detection, retention time correction and alignment. The metabolites were identified by accuracy mass (< 25 ppm) and MS/MS data which were matched with a standard database. In the extracted-ion features, only the variables having more than 50% of the nonzero measurement values in at least one group were kept.

### Statistical analysis

Replicate was treated as the experimental unit in this study. Statistical analysis for all parameters except metabolomics data was performed with Statistical Package for the Social Sciences (SPSS) 19.0 (IBM, Armonk, USA). Independent-samples *t*-test was performed to test the significant mean differences between groups. All results were presented as means and pooled standard errors of means (SEM). A probability of *p* < 0.05 was considered significant.

For metabolomics data, SIMCAP software (Version 14.0, Umetrics, Umeå, Sweden) was used for multivariate data analysis and modeling. Data were mean-centered using Pareto scaling. Models were built on orthogonal partial least-square discriminant analysis (OPLS-DA). All the models evaluated were tested for over fitting with methods of permutation tests. OPLS-DA allowed the determination of discriminating metabolites using the variable importance on projection (VIP), that VIP > 1 was considered as significant, and higher VIP was in agreement with a strong discriminatory ability for potential biomarker selection. The discriminations of metabolites between two groups were obtained using a statistically significant threshold of VIP value from OPLS-DA model and *p* value from two-tailed Student’s *t* test, that VIP value > 1.0 and *p* value < 0.05 were considered significant. Fold change was calculated as the logarithm of the average mass response ratio between two arbitrary classes. To identify the altered biological pathways, the differential metabolite data were performed KEGG pathway analysis using KEGG database (http://www.kegg.jp). KEGG enrichment analysis was conducted with the Fisher’s exact test. Enriched KEGG pathway was considered statistically significant at the *p* < 0.05 level.

## Results

### Production performance

Results of production performance associated parameters are shown in Table 2. EGCG significantly increased the production rate of ducks (*p* = 0.014). However, no differences were found in qualified egg rate, average egg weight, daily egg yield, F/E, or mortality between ducks in two treatments (*p* > 0.050).

**Table 2.** Effects of EGCG on production performance of laying ducks reared in high temperature condition^1^.

Item	CON	EGCG	*p* value
Production rate (%)	66.86 ± 3.28	73.54 ± 3.48	0.014
Qualified egg rate (%)	98.91 ± 1.29	99.33 ± 0.71	0.526
Average egg weight (g)	71.12 ± 1.97	69.86 ± 2.72	0.427
Daily egg yield (g)	47.53 ± 2.24	53.29 ± 5.83	0.079
F/G	2.69 ± 0.09	2.65 ± 0.26	0.758
Mortality (%)	1.04 ± 2.55	1.04 ± 2.55	1.000

CON, control group; EGCG, epigallocatechin gallate; F/G, feed gain ratio.

^1^Results were based on 6 replicates per treatment.

### Egg quality

Results of egg quality are shown in Table 3. EGCG showed no significant effects on shell strength (*p* = 0.879), shell thickness (*p* = 0.426), yolk color (*p* = 0.232), or Haugh unit (*p* = 0.551) of eggs laid by laying ducks in the two groups, which implied that dietary EGCG had no effect on egg quality of laying ducks under high temperature condition.

**Table 3.** Effects of EGCG on egg quality of laying ducks reared in high temperature condition^a^.

Item	CON	EGCG	*p* value
Egg shell strength (N)	55.70 ± 7.96	56.37 ± 11.20	0.879
Egg shell thickness (mm)	0.389 ± 0.015	0.397 ± 0.286	0.426
Yolk color	3.90 ± 0.32	3.60 ± 0.70	0.232
Haugh unit	69.48 ± 14.00	73.22 ± 11.97	0.551

CON, control group; EGCG, epigallocatechin gallate.

^a^ Results were based on 24 samples per treatment.

### Serum immune status

The effect of dietary EGCG on serum immune status associated indexes is shown in Table 4. EGCG significantly increased the IgA (*p* = 0.008) and IgG (*p* = 0.006) levels in serum of laying ducks under high temperature. But no differences were found in serum IgM level between two groups (*p* = 0.124).

**Table 4.** Effects of EGCG on hepatic immune status of laying ducks reared in high temperature condition^a^.

Item	CON	EGCG	*p* value
IgA (μg/mL)	42.34 ± 4.51	49.92 ± 6.74	0.008
IgG (μg/mL)	303.28 ± 31.28	368.49 ± 58.22	0.006
IgM (μg/mL)	83.87 ± 6.07	90.67 ± 11.86	0.124

CON, control group; EGCG, epigallocatechin gallate; IgA, immunoglobulin A; IgG, immunoglobulin G; IgM, immunoglobulin M.

^a^Results were based on 12 samples per treatment.

### Hepatic antioxidant capacity

The effect of dietary EGCG on hepatic antioxidant capacity associated indexes are shown in Table 5. EGCG significantly increased the activities of SOD (*p* = 0.012), CAT (*p* = 0.009), GSH-Px (*p* = 0.021), and level of HSP-70 (*p* = 0.003) in the liver of laying ducks under high temperature condition. But no differences were found in MDA content (*p* = 0.890) or T-AOC (*p* = 0.239) in the liver of laying ducks between two groups.

**Table 5.** Effects of EGCG on hepatic antioxidant status of laying ducks reared in high temperature condition^a^.

Item	CON	EGCG	*p* value
SOD activity (U/mL)	1.16 ± 0.19	1.43 ± 0.23	0.012
MDA content (nmol/mL)	1.34 ± 0.35	1.32 ± 0.31	0.890
CAT activity (U/mL)	102.70 ± 15.74	123.96 ± 16.87	0.009
T-AOC (mmol/L)	0.43 ± 0.08	0.39 ± 0.09	0.239
GSH-PX activity (U/mL)	2.11 ± 0.31	2.54 ± 0.44	0.021
Hsp-70 (pg/mL)	50.20 ± 5.96	60.26 ± 7.35	0.003

CON, control group; EGCG, epigallocatechin gallate; SOD, superoxide dismutase; MDA, malonaldehyde; CAT, catalase; T-AOC, total antioxidant capacity; GSH-Px, glutathione peroxidase; Hsp-70, heat shock protein-70.

^a^ Results were based on 12 samples per treatment.

### Hepatic metabolomics

**Figure 1.**
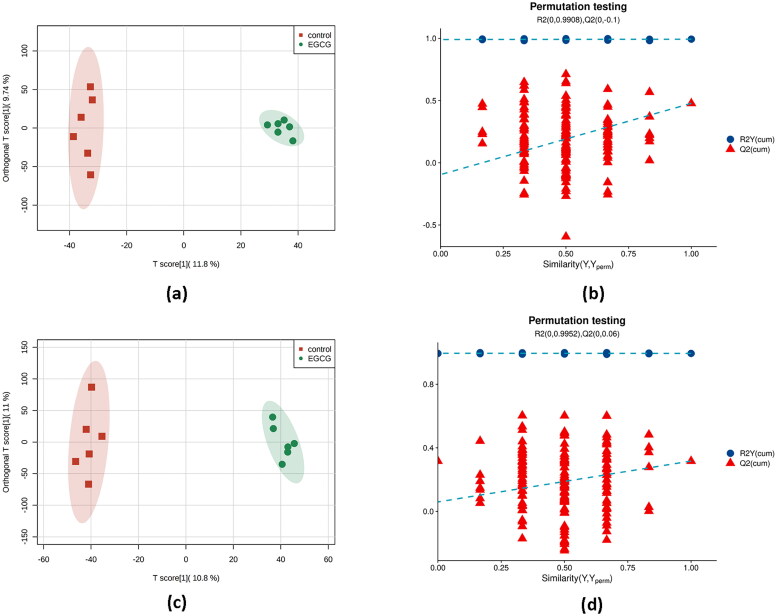
Plots of multivariate statistical comparison of hepatic metabolome data in ducks between CON and EGCG groups. (a) OPLS-DA score plot in negative ion mode; (b) permutation test on OPLS-DA plot in negative ion model; (c) OPLS-DA score plot in positive ion mode; (d) permutation test on OPLS-DA plot in positive ion model.

**Figure 2.**
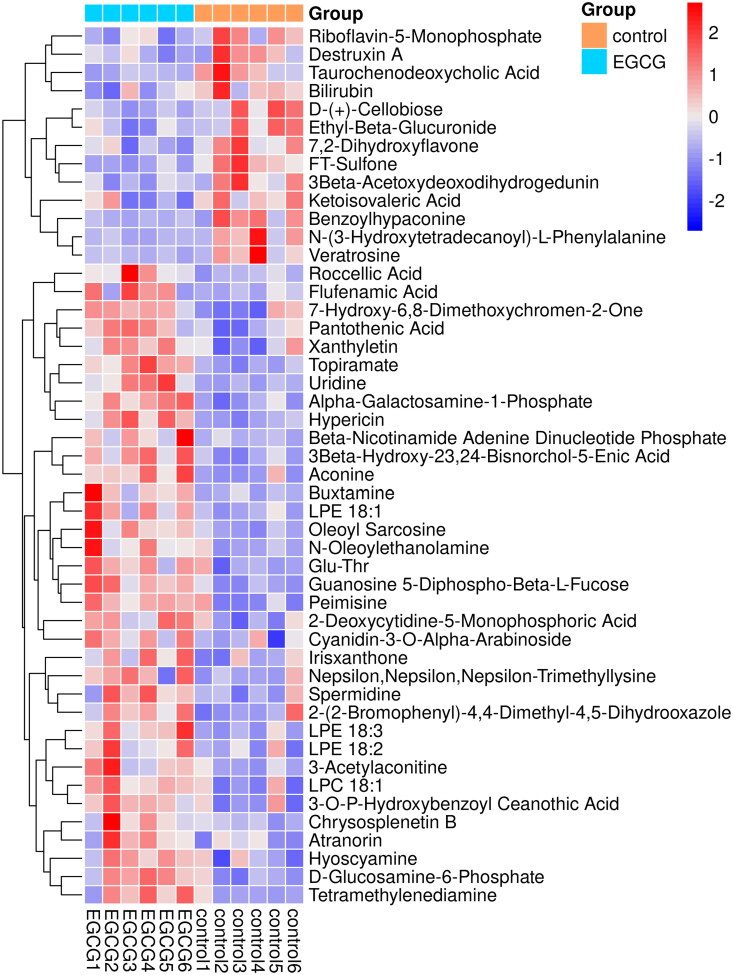
Hierarchical clustering of differential hepatic metabolites in ducks between CON and EGCG groups.

Score plots of OPLS-DA in negative and positive ion models (Figure 1a, c) showed clear discriminations between hepatic metabolites of ducks in two groups without any overlap (*R*^2^X = 21.5%, *R*^2^Y = 99.4%, *Q*^2^ = 47.8%), demonstrating a robust metabolic difference between the two groups. Validation using permutation test indicated that the models had not been overfitted (Figure 1b, d). A total of 48 differential metabolites were obtained based on VIP > 1 derived from OPLS-DA model and *p* < 0.05 in *t*-test, of which 13 metabolites were down-regulated and 55 metabolites were up-regulated in the liver of laying ducks in EGCG group compared to the ones in CON group (Figure 2).

**Figure 3.**
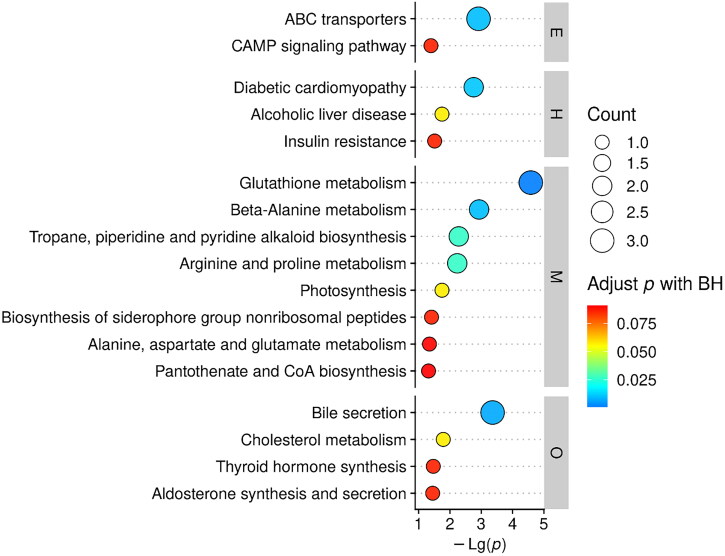
KEGG pathway analysis overview of altered hepatic metabolic pathways in ducks between CON and EGCG. Lg10 (P) indicates log operation on the ion abundance of metabolites identified by mass spectrometry. Each dot represents a pathway and the color indicates the *p*-value of the enrichment. A larger circle indicates that there are more metabolites annotated to this pathway. E is environmental information processing. H is human diseases. M is metabolism. O is organismal systems.

**Figure 4.**
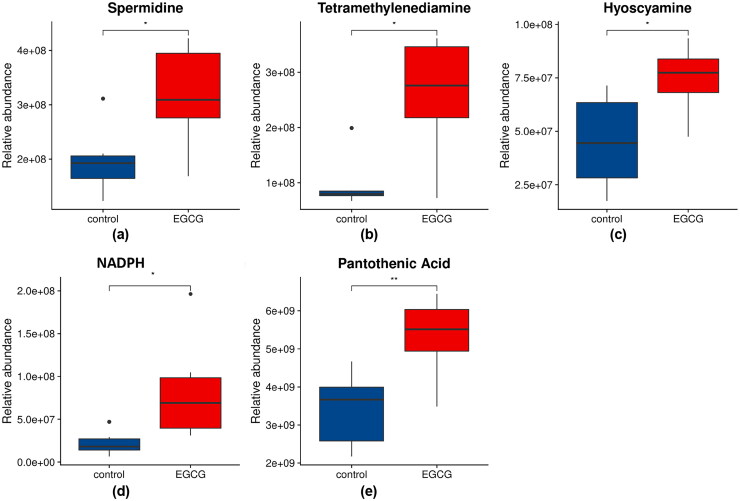
Relative abundances of metabolites involved in the 4 significantly altered metabolic pathways. (a) Spermidine; (b) tetramethylenediamine; (c) hyoscyamine; (d) β-nicotinamide adenine dinucleotide phosphate (NADPH); (e) pantothenic acid.

KEGG pathway analysis of the differential metabolites is shown in Figure 3. Pathways are classified into environmental information processing associated pathways (represented by E), human diseases associated pathways (represented by H), metabolic pathways (represented by M), and organismal systems associated pathways (represented by O). In metabolic pathways, a total of 4 metabolic pathways were shown to be significantly associated to the effect of EGCG on laying ducks raised in high temperature (adjust *p* value < 0.05), which were arginine and proline metabolism, tropane, piperidine, and pyridine alkaloid biosynthesis, glutathione metabolism, and β-alanine metabolism. For arginine and proline metabolism, 2 metabolites were found involved, which were spermidine and tetramethylenediamine (Figure 4a, b). For tropane, piperidine, and pyridine alkaloid biosynthesis, 2 metabolites were found involved, which were tetramethylenediamine and hyoscyamine (Figure 4b, c). For glutathione metabolism, 3 metabolites were found involved, which were spermidine, tetramethylenediamine, and β-nicotinamide adenine dinucleotide phosphate (NADPH) (Figure 4a, b, d). For β-alanine metabolism, 2 metabolites were found involved, which were spermidine and pantothenic acid (Figure 4a, e). Moreover, the concentrations of the metabolites involving in the 4 significantly altered metabolic pathways were all significantly higher in the hepatic tissue of laying ducks in EGCG group compared to the ones in CON group (Figure 4, p < 0.050).

## Discussion

*Linwu* duck is an important indigenous duck breed for both meat and egg purposes in China, with the characteristics of fast growth, high egg production, high feed efficiency, and unique meat flavor and texture. Heat stress is one of notorious factors that diminishes both growth and production performances of ducks, and causes tremendous economic losses to the duck breeders (He et al. 2019; Chen et al. 2021). Apart from the common strategies such as establishments of fans and cooling pads in facilities, supplementing phytochemical substance in feed could be another way to fight against high temperature in poultry farming. Previous studies proved that EGCG used as feed additives in poultry achieved promising results. EGCG supplemented at 600 mg/kg in diet increased the body weight gain and feed intake of 35-d-old broilers which were exposed to high temperature (Xue et al. 2017). Dietary EGCG at level between 0 and 400 mg/kg linearly improved the feed intake and egg production of quails reared in high temperature condition, possibly due to the fact that EGCG prevented lipid peroxidation and enhanced antioxidant defense system *via* increasing hepatic NF-κB expression (Sahin et al. 2010). In our study, 300 mg/kg EGCG supplemented in diet increased the egg production rate of laying ducks under high temperature, which was in line with the previous reports. However, no effects were found on eggshell strength and thickness, yolk color, or Haugh unit with the dietary supplementation of 300 mg/kg EGCG. This was partially supported by an earlier study by Wang et al. (2020), who reported that diet supplement of 165 mg/kg EGCG had no effects on eggshell strength, eggshell thickness, or yolk color, but improved Haugh unit of eggs produced by 35-w-old Lohmann laying hens. However, another study claimed that dietary supplementation with 0.3–1.0% green tea water extracts (contains 8.4% EGCG in the extract product) decreased eggshell thickness and eggshell strength of 20-w-old Xianju laying hens (Xia et al. 2022). The inconsistent results of EGCG’s effects on egg quality might be due to the different poultry varieties, EGCG levels, and physiological statuses of experimental animal used in studies.

Immunoglobulins are essential molecules for the avian adaptive immune response, which are produced by lymphocyte cells in birds and served as the first line to defense the intruders (Berghof et al. 2018). High temperature suppresses both cell mediated and humoral immune responses, leading to alterations of immunoglobulin synthesis (Hu et al. 2022). Previous studies found that EGCG could alleviate stress that indued immune suppression in animals. Liu et al. (2021) reported that EGCG promoted the immune function of ileum in high fat diet-fed mice by regulating immunoglobulin production. Another study by Yan et al. (2021) also found that EGCG showed protective effect on mice by increasing the level of IgA, T cells, and neutrophils against *Acinetobacter baumannii* infection induced stress. Similar with these results, we found that under high temperature, the laying ducks fed with 300 mg/kg EGCG supplemented in diet had increased serum levels of IgA and IgG compared to the ones fed with basal diet. It suggested that EGCG improved the adaptive immune status of laying ducks reared in high temperature, which could potentially contribute to the EGCG’s effect on improving egg production performance.

Liver is an important organ in bird that maintains nutritional homeostasis, produces circulatory proteins, coordinates transitions related to adaptation in carbohydrate, lipid, and protein metabolism (Zhang et al. 2021). Hepatic antioxidants consist of antioxidative enzymes, and defensive proteins such as heat shock proteins (Hsps) (Surai et al. 2019). Their levels reflect the antioxidative capability of the body system, and provide valuable information of organism’s health condition. Generally, high temperature increases synthesis or ROS production, causes oxidative damage to cellular lipids and proteins, and eventually overcomes the antioxidative defense system (Yang et al. 2010). EGCG as a kind of polyphenol shows high antioxidative effect, which is a potential attenuator for the negative effects of high temperature in poultry production (Zhao et al. 2021). Previous studies found that dietary inclusion of EGCG improved the body weight and level of antioxidant enzymes such as GSH-Px, SOD, and CAT in the liver and serum of broilers in high temperature (Xue et al. 2017). Diet supplemented with 200 or 400 mg/kg EGCG significantly increased feed intake, hepatic SOD, CAT, and GSH-Px activities, and decreased hepatic MDA level of female quails (Sahin et al. 2010). Yin et al. (2021) also found that tea polyphenols induced higher expression of Hsp-70 in H9C2 cells in high temperature conditions (42 °C). In line with these studies, we found in the present study that 300 mg/kg EGCG increased the hepatic activities of SOD, CAT, GSH-Px, and content of Hsp-70 in *Linwu* laying ducks, proving the antioxidative effect of EGCG by enhancing the antioxidative enzyme activity and Hsps expression. However, hepatic MDA level was not changed with the dietary EGCG, which was possibly due to the reason that the high temperature caused acute oxidative injury in layers that overcame the eliminating capabilities of the increased antioxidant enzymes by EGCG (Tan et al. 2010). A possible pathway that EGCG regulated the antioxidative enzyme activities and Hsps expression could be the transcription factor nuclear factor (erythroid-derived 2)-like 2 (Nrf2) pathway, and Nrf-2 further activated regulated heme oxygenase-1 (HO-1) and Hsp70 expression (Naidu et al. 2015; Paul et al. 2018; Hu et al. 2019).

**Figure 5.**
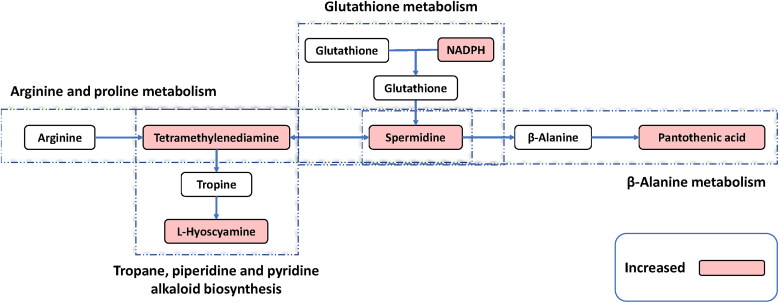
Schematic diagram of the modulated metabolites and potential disturbed metabolic pathways. Up-regulated metabolites detected are shown in the red background; blank background means no statistically significant change or undetected. NADPH, β-nicotinamide adenine dinucleotide phosphate.

In order to interpret the metabolic mechanism of the protection effects of EGCG on laying ducks under high temperature condition, hepatic metabolomics was conducted in the present study. By comparing the omics data, 68 differential metabolites (13 downregulated/55 upregulated) were identified in ducks of EGCG group compared to CON. Arginine and proline metabolism, tropane, piperidine, and pyridine alkaloid biosynthesis, glutathione metabolism, and β-alanine metabolism were the 4 most significantly altered metabolic pathways in laying ducks with 300 mg/kg EGCG treatment. A schematic diagram of the modulated metabolites and potential disturbed metabolic pathways is shown in Figure 5.

Arginine and proline metabolism was reported to play an important role in relieving inflammation, since up-regulation of arginine and proline metabolism dampened the intestinal inflammation during *Salmonella* infection in chicken (Mon et al. 2020). Raza et al. (2020) stated that stimulation of arginine and proline metabolism pathway by gut symbionts (*K.michiganensis* BD177) increased the flies’ resistance to low-temperature stress. In chicken, arginase and proline metabolism contributed to polyamines synthesis, including tetramethylenediamine and spermidine (Furukawa et al. 2021). These polyamines are important in modulating gene expression, cell growth and proliferation, protein translation, cell apoptosis, and could act as antioxidant molecules (Castro and Kim 2020). A study in weaning piglets showed that 0.5–0.15% tetramethylenediamine supplemented in diet improved organs’ physical barrier function and antioxidant capacity in dose- and tissue-dependent manner (Liu et al. 2019). Besides, dietary spermidine was proved to prevent ethanol and lipopolysaccharide-induced hepatic fibrosis, attenuated the hepatic oxidative stress by decreasing the levels of reactive oxygen species and lipid peroxidation, and prevented the expression of pro-inflammatory cytokines by blocking the phosphorylation of the inhibitory protein (Adhikari et al. 2021). In the present study, hepatic contents of tetramethylenediamine and spermidine in laying ducks reared under high temperature condition were increased by dietary EGCG. According to previous researches, these polyamines might contribute to the antioxidant and anti-inflammation effects of EGCG. Therefore, EGCG promoting arginine and proline metabolism pathway, stimulating the biosynthesis of tetramethylenediamine and spermidine could be one potential metabolic mechanism of EGCG’s protection effect on laying ducks under high temperature.

On the other hand, tetramethylenediamine could be converted into l-hyoscyamine in tropane, piperidine and pyridine alkaloid biosynthesis pathway. A previous study reported that hyoscyamine showed antioxidant and cytoprotective action, since hyoscyamine reduced lipid peroxide of vital organs, stabilized biomembrane, protected cell structure, and maintained cell function (Zhan et al. 2007). In our study, EGCG also increased the hepatic level of hyoscyamine in laying ducks, which might help attenuating the antioxidant and immunological stresses caused by high temperature.

Another significantly altered metabolic pathway associated with EGCG functioning on laying ducks under high temperature was glutathione metabolism, in which glutathione disulfide was transformed to glutathione with the catalyzation of GSH-Px and glutathione reductase in the presence of NADPH (Partyka and Niżański 2021). In our study, hepatic NADPH level in laying ducks under high temperature condition was increased by dietary EGCG, which might contribute to the biosynthesis of glutathione. Glutathione is considered the most abundant molecules controlling redox balance and signaling, regulating transcription factors and gene expression, and many other important cellular pathways (García-Giménez et al. 2017). Therefore, another potential mechanism of the antioxidant effect of EGCG on laying duck reared in high temperature condition could be EGCG increased the hepatic content of NADPH, which participates in glutathione metabolism pathway and enhances the production of glutathione. However, this hypothesis needs further investigation due to the lack of direct evidence to clarify the increased content of glutathione in the liver of duck under the effect of EGCG in the present study.

At last, the hepatic β-alanine metabolism pathway in laying ducks under high temperature condition was identified being significantly changed under the effect of dietary EGCG. In β-alanine metabolism pathway, spermidine could induce the production of pantetheine and activate the biosynthesis of pantothenate and coenzyme A (CoA) (Jiang et al. 2023). Pantothenic acid, as a type of vitamin B, is prosthetic group of coenzyme A and a part of acyl carrier proteins, and is involved in the metabolism of carbohydrates, fats, and proteins in the body (Zhang et al. 2023). Pantothenate enhanced the efficacy of anti-PDL1 antibody therapy in murine tumor model, and was positively correlated with the response to anti-PD1 therapy in patients with melanoma, which implied that pantothenate drove T cell polarization, bioenergetics, and immune capability (St Paul et al. 2021). Additionally, dietary supplementation with pantothenic acid improved growth performance and antioxidant status of white Pekin ducks (Tang et al. 2020). In the present study, hepatic content of pantothenic acid was increased by dietary EGCG, along with increased antioxidant and immune capability in the ducks. It suggested that a possible explanation for the protection effect of EGCG is that EGCG activated the β-alanine metabolism pathway and induced the production of pantothenic acid, which improved the antioxidant and immune capability of the laying ducks reared in high temperature condition.

## Conclusion

In conclusion, supplementation of 300 mg/kg EGCG in diet improved egg production rate of laying ducks suffering high temperature, and strengthened the antioxidant and immune capacities of ducks by increasing hepatic antioxidative enzyme capacities and Hsp-70 level. Metabolomics analysis revealed that EGCG influenced the pathways of arginine and proline metabolism, tropane, piperidine, and pyridine alkaloid biosynthesis, β-alanine metabolism, and glutathione metabolism pathways, and the altered metabolites might contribute to antioxidant and immune enhancement to the laying duck. These findings provided potential clues for the underlying mechanisms of EGCG’s protection effects on laying ducks reared in high temperature condition.
